# Development of a Novel Spinneret Design for Improved Melt Extrusion Performance: A Computational and Empirical Study

**DOI:** 10.3390/polym18010115

**Published:** 2025-12-30

**Authors:** Nereida Guadalupe Ortiz-Leyva, Giuseppe Romano, Jack Wilson, Jonathan C. Hunter, Alessandro De Rosis

**Affiliations:** 1 Fibre Extrusion Technology Ltd., Gelderd Road, Leeds LS27 7JU, UK; nereida.ortiz@fetuk.com (N.G.O.-L.); jack@fetuk.com (J.W.); 2 Department of Mechanical and Aerospace Engineering, The University of Manchester, Manchester M13 9PL, UK; giuseppe.romano@manchester.ac.uk

**Keywords:** polymer extrusion, non-Newtonian fluids, parametric study, computational fluid dynamics (CFD), spinneret design, extrusion efficiency

## Abstract

This study presents a comprehensive evaluation of a novel spinneret design to enhance polymer melt extrusion performance in fibre spinning production. Computational fluid dynamics (CFD) simulations using ANSYS Polyflow 2024 R2 are employed to analyse flow behaviour, pressure distribution, and shear profiles within the die. The novel design demonstrates improved flow uniformity, reduced pressure fluctuations, and minimized high-shear regions compared to a baseline spinneret. Experimental validation is conducted through side-by-side extrusion tests using polypropylene and thermoplastic polyurethane, confirming the simulation results. Throughput efficiency tests further reveal that the novel spin pack design significantly reduces residence times by 16% and accelerates purging cycles, indicating fewer polymer stagnation zones and enhanced material changeover efficiency. The computational parametric study conducted on PP shows that the novel design demonstrates improved flow uniformity and a significant reduction in operating pressure, achieving an 11% decrease in die-head pressure compared to the baseline spinneret. Additionally, the optimized geometry successfully minimizes high-shear regions while maintaining a manageable maximum shear rate increase of approximately 19% at the walls, which aids in preventing wall slip. These enhancements lead to lower extrusion pressures and more consistent processing across various polymers. By minimizing material waste and improving process reliability, the new spinneret design contributes to a more sustainable, cost-effective manufacturing process. Overall, these improvements provide a valuable framework for advancing extrusion technologies and optimizing spinneret geometries for high-performance polymer extrusion. The novelty of this work lies in introducing a spinneret geometry specifically optimized to minimize melt residence time, an outcome directly linked to reduced material degradation and waste.

## 1. Introduction

**Figure 1:**
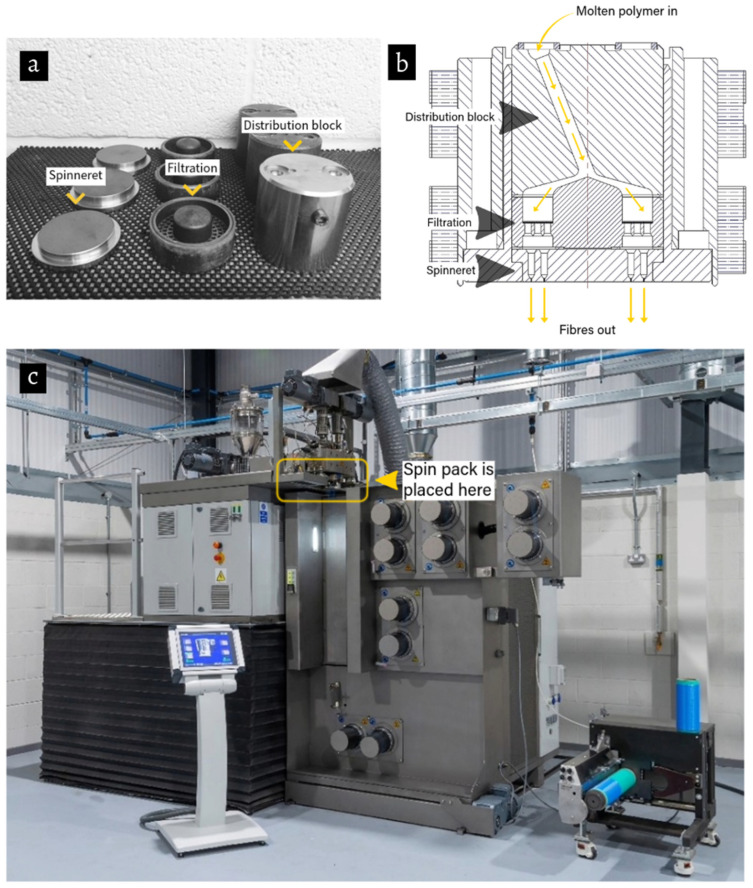
(**a**) Photograph of typical parts comprising a standard spin pack. (**b**) Schematic of a cross-sectional view of a standard spin pack. (**c**) Photograph illustrating where the spin pack is placed on melt extrusion equipment (courtesy of FET Ltd., Leeds, UK [1]).

Polymer extrusion by melt spinning is a well-established manufacturing technique extensively utilised across the textile, plastics, food, and pharmaceutical industries for the production of advanced functional materials. In this process, a molten polymer is conveyed through a specifically designed die, the geometry of which dictates the cross-sectional profile of the resulting extrudate. Central to the fibre extrusion system is the *spin pack*, a critical component responsible for distributing the molten polymer into multiple, precisely controlled streams to form individual filaments. The general configuration of a conventional spin pack is illustrated in Figure 1, which shows its different elements, a cross-sectional schematic, and its placement within a melt-spinning system.

**Figure 2:**
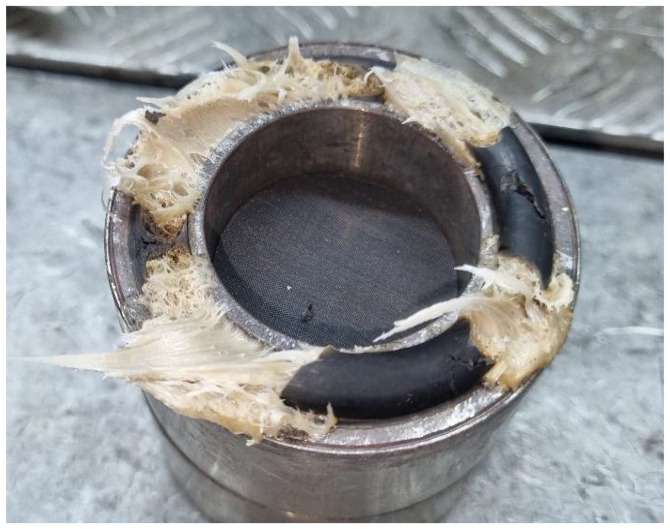
Photograph illustrating degraded polymer within a spin pack (courtesy of FET Ltd., Leeds, UK [1]).

The design of the spinneret is a determining factor in achieving uniform flow and consistent product quality during polymer extrusion. Inadequate spinneret geometry can induce non-uniform flow distribution, excessive pressure drops, and the formation of stagnant regions, commonly referred to as *dead zones*. These regions promote polymer accumulation and prolonged residence times, leading to thermal degradation of the material when exposed to elevated temperatures (typically above 200 °C—see Figure 2). Such degradation manifests as discolouration, surface irregularities, or filament breakages, ultimately resulting in non-conforming yarn and reduced process reliability.

Fibre extrusion industries, such as FET UK Ltd., typically achieve process material efficiencies exceeding 99% when processing common polymers, including polypropylene (PP), polyethylene terephthalate (PET), and nylon. However, efficiency often declines when processing biodegradable polymers, such as polydioxanone (PDO) and polylactic acid (PLA), or high-performance polymers, including polyether ether ketone (PEEK) and polycarbonate (PC). Addressing these challenges would enable the production of complex polymers with manufacturing efficiencies comparable to those of general-purpose materials.

Optimisation of spinneret design and polymer flow behaviour has been the subject of numerous investigations; however, much of the existing literature focuses on processing techniques such as electrospinning [2,3,4], and dry-jet wet spinning [5,6,7,8], with comparatively few studies addressing melt extrusion applications. Further advancements in spinneret geometry and flow control within the spin pack are critical, as these parameters directly govern the uniformity and mechanical quality of extruded fibres.

Early research into spinneret design dates back to the late 1970s, when Kirichenko et al. [9] examined the influence of die geometry on the physical–mechanical properties of Capron yarns produced via demonomerized polymer melt spinning. Their work compared rectangular spinnerets with parallel hole arrays to conventional circular designs with concentric holes, demonstrating that rectangular geometries yielded 1.2–1.7 times more uniform cooling conditions.

Traditionally, spinneret optimisation has relied heavily on empirical testing and iterative prototyping, both of which are time-consuming and cost-intensive. In recent decades, however, computational fluid dynamics (CFD) tools such as ANSYS Polyflow 2024 R2, tailored specifically for polymer processing, have transformed spinneret design methodologies. These tools enable accurate prediction of pressure, velocity, and shear rate distributions within complex spinneret geometries, thereby facilitating quantitative evaluation of design modifications before fabrication.

Through systematic numerical simulation, engineers can refine spinneret geometry to achieve consistent flow uniformity and mitigate detrimental effects such as dead zones or excessive shear stresses that lead to polymer degradation [10]. The emergence of CFD-based optimisation has therefore provided a rigorous, cost-effective framework for design development, allowing for rapid prototyping and performance prediction without extensive experimental trials. Several studies have demonstrated the value of such approaches; for instance, modifications in entry angle, hole arrangement, or incorporation of multi-layer structures have been shown to reduce vortex formation and enhance flow symmetry [11].

Qin et al. [12] conducted a numerical investigation to understand the effects of the geometrical parameters of the spinneret orifice (such as the length-to-diameter ratio and taper angle) on the UHMWPE melt flow stability. Xia et al. [8] employed numerical simulation to optimise the spinneret design for dry-jet wet spinning of cellulose/[bmim]Cl solutions, demonstrating that an increased aspect ratio reduced apparent viscosity, while smaller entrance angles minimised the formation of stagnant regions. Similarly, Karuppasamy et al. [13] reported that optimised conical spinnerets with smoother entry angles effectively reduced flow instabilities and improved polymer chain alignment, thereby enhancing mechanical performance.

Despite these advances, empirical validation of novel spinneret geometries under industrial melt extrusion conditions remains scarce. Widjojo et al. [14] combined CFD simulations with experimental testing to design spinnerets that mitigated die swell and instability in hollow hyperbranched polyethersulfone (HPES) fibres, achieving reduced extrudate distortion and enhanced process stability. More recently, Moriam et al. [15] systematically investigated the influence of geometric parameters (such as hole diameter, density, entrance angle, and length-to-diameter ratio) on the mechanical performance of Ioncell fibres produced by dry-jet wet spinning, combining experimental testing and numerical simulation. Their research involved a combination of experimental testing and numerical simulations, varying several factors: the diameter of the spinneret holes, hole density, hole distribution (both rectangular and circular), entrance angles (8° and 60°), and the length-to-diameter ratio (L/D) of the holes. The results indicated that a rectangular spinneret with an 8° entrance angle and an L/D ratio of 1 produced fibres with the highest toughness.

Recent advancements in polymer processing have emphasized the importance of spinneret and die design in improving melt flow behavior, reducing dead zones, and enhancing overall extrusion efficiency. CFD-based studies have shown that modifications to spinneret geometry, such as entry angle and flow channel design, directly affect velocity distribution and stagnation zones [13,16]. Leithäuser et al. [16] applied shape optimization to eliminate dead zones and reduce residence time in spin pack geometries. Furthermore, recent developments in automated die optimization using computational frameworks, such as the approach by Wagner et al. [17], have enabled simultaneous balancing of outlet flow and reduction in pressure drop. Accurate simulation of these behaviours depends strongly on validated rheological models, as outlined by Urraca et al. [18], who confirmed the Bird–Carreau model’s accuracy through experimental pressure drop comparisons. Comprehensive reviews by Vergnes [19] have also reinforced the significance of residence time distribution in extrusion performance. These studies support the critical role of simulation-guided design in minimizing stagnation, improving throughput, and advancing sustainable polymer processing systems.

Nonetheless, dedicated studies addressing the interplay between spinneret and spin pack geometries in melt extrusion remain limited. In particular, systematic approaches to minimising dead zones and improving melt flow uniformity in multifilament yarn extrusion are rare. The present work addresses this gap by employing numerical simulations to evaluate a range of spinneret configurations, aiming to identify optimal geometric features that balance pressure, velocity, and shear rate distributions. These findings are subsequently validated through targeted experimental testing, providing a comprehensive framework for the design of high-efficiency spinnerets that enhance flow uniformity, reduce pressure fluctuations, and improve overall extrusion performance. The key novelty of this study lies in the design of a new spinneret configuration using ANSYS Polyflow 2024 R2 that reduces the molten polymer’s residence time, thereby lowering material waste and improving manufacturing performance. This is particularly critical for medical-grade and high-performance polymers, where thermal exposure and degradation must be rigorously controlled.

## 2. Modelling Details

### 2.1. Simulation Set-Up

In this study, ANSYS Polyflow 2024 R2 is employed to simulate the behaviour of different polymers as they pass through a range of spinneret designs under steady-state operating conditions. The Carreau–Yasuda model is incorporated to capture shear-thinning behaviour, while the Arrhenius law accounts for the temperature dependence of the non-Newtonian polymer melt. Key parameters, including geometry, hole count, and temperature, are varied in a systematic manner to assess their influence on the flow field.

**Figure 3:**
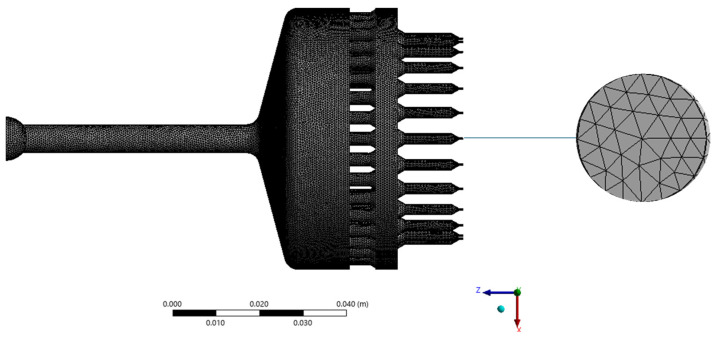
The mesh used for model D0 was adopted for all other models, maintaining the same mesh type and size.

The flow boundary conditions are selected to represent realistic extrusion settings, with prescribed inlet flow rates, specified outlet pressures, and no-slip walls throughout the spinneret. A fine computational mesh is generated to resolve the intricate flow features within the spin pack; refinement is concentrated around critical regions such as the capillary holes and connecting channels to ensure numerical stability and accuracy. A predominantly hexahedral mesh with a characteristic size of 0.001 m is used, as shown in Figure 3. A mesh sensitivity analysis was performed to ensure grid independence of the numerical results. Four meshes with characteristic element sizes of 1 × 10^−2^ m, 5 × 10^−3^ m, 1 × 10^−3^ m, and 5 × 10^−4^ m were tested. Die-head pressure, maximum shear rate, and average outlet velocity were monitored as key output variables. As the mesh was refined from 1 × 10^−2^ m to 1 × 10^−3^ m, the predicted pressure decreased from 3.06 MPa to 2.92 MPa, while shear rate and velocity variations progressively diminished. Further refinement to 5 × 10^−4^ m resulted in changes below 5% for all monitored quantities, indicating grid-independent behaviour. Based on this criterion, the mesh with a characteristic size of 1 × 10^−3^ m was selected as the baseline mesh for all simulations, providing an optimal balance between numerical accuracy and computational cost. The simulations are performed using a steady-state solver, and convergence is assessed through reductions in residuals and stabilisation of key flow variables, including velocity and pressure. To ensure consistency in the viscosity field, a Picard iteration scheme is applied throughout the solution process.

### 2.2. Temperature Dependency

The dependence of the polymer viscosity *η* on the temperature *T* is modelled via the Arrhenius law [20] in Equation (1), that is

(1) $$\eta \left(T\right)=\eta_{Ref}\times e\frac{E_{a}}{RT}\;,$$

where, in Equation (1), *E_a_* is the activation energy (J/mol), *η_Ref_* is the viscosity at reference temperature (Pa∙s), and *R* is the universal gas constant (8.314 J/mol∙K).

### 2.3. Shear Thinning

The Carreau–Yasuda law given in Equation (2) is used to describe the non-Newtonian behaviour of polymers during extrusion through the spinneret. It accounts for the shear-thinning nature of many polymers, where the viscosity decreases with increasing shear rate. This law is widely applied in polymer processing to model the viscosity behaviour of shear-thinning fluids [21]. The mathematical expression reads as follows:

(2) $$\eta =\eta_{\infty }\alpha_{T}+\alpha_{T}\left(\eta_{0}-\eta_{\infty }\right)\;{\left[1+{\left(\lambda \alpha_{T}\dot{\gamma }\right)}^{a}\right]}^{\frac{n-1}{a}},$$

where, in Equation (2), η_0_ is the zero-shear viscosity (Pa∙s), which is the viscosity as the shear rate approaches zero, η_∞_ is the infinite-shear viscosity (Pa∙s), representing the viscosity when the shear rate tends toward infinity, $\dot{\gamma }$ is the effective shear rate (s^−1^), i.e., the shear rate applied to the fluid, *λ* is the time constant (s), influencing the nonlinear behaviour, *n* is the power-law index (dimensionless), defining the degree of shear thinning, *a* is the transition parameter (dimensionless), controlling the smoothness of the transition, and *α*_T_ is the temperature-dependence factor (dimensionless), adjusting viscosity based on temperature. The rheological parameters used for the PP melt are reported in Table 1, and are taken from Ansys Polyflow 2024 R2.

### 2.4. Design Enhancement Study

A design enhancement study is carried out to investigate how different combinations of spinneret geometries and hole counts influence extrusion performance. For each configuration, the resulting flow distribution, pressure drop, and shear rates are assessed to identify the most effective design and to understand the specific advantages offered by each variation. Several geometric modifications are explored with the aim of improving flow uniformity and reducing pressure losses, while the number of holes is systematically varied to examine its impact on both throughput and the consistency of the extruded filaments.

The resulting simulation data, including pressure contours, velocity fields, and shear rate distributions, are analysed holistically to determine the configuration that offers the best balance of uniform flow, manageable pressure requirements, and favourable shear characteristics. This assessment informs the selection of the optimal combination of design parameters capable of delivering enhanced spinneret performance.

We investigate the configurations listed below:

D0: Initial
D1: Kinked entry high
D2: Bi-Co version of deep entry
D3: Reduced volume filter
D4: Double layer (48 holes) spinneret
D5: Inverted dome
D6: No dome
D7: Offset entry
D8: Smaller dome size
D9: Pyramid shape
D10: Rounded edges
D11: Wider cone
D12: Lipped cone
D13: Cooler spinneret
D14: Doughnut cone
D15: Kinked entry low

All designs are tested with a volumetric flow rate at the inlet of 8.333 × 10^−8^ m^3^/s. The models operate under isothermal conditions (i.e., T = 220 °C), except for D13, which had the end part of the spinneret at 200 °C. The material properties used in the simulations are the default isothermal and non-isothermal polypropylene (PP) available in ANSYS Poly flow 2024 R2. The PP density used is 1100 kg/m^3^.

## 3. Extrusion Experimentation

### 3.1. Melt Extrusion

Side-by-side testing is conducted to validate the numerical simulation results using a final design (DF) that integrated features from the best spinneret designs. This new design is compared against the baseline design (D0). Two main tests are conducted during this phase of the experimental work. In the former, polypropylene (PP) sourced by Ineos (London, UK) (100-GA03, MFI 3) is evaluated on a 25 mm Ø (30 L/D) extruder (FET general-purpose screw design), with a metering pump capacity of 1 cc/rev. In the latter, thermoplastic polyurethane (TPU), sourced by Covestro (Leverkusen, Germany) (DESMOPAN 2795A SMP, MFI 17), is tested on a 20 mm Ø (30 L/D) extruder.

The process starts by setting the extruder temperature to 200 °C. The polymer output is set at 5, 15, and 25 mL/min for this test. Depending on the polymer temperature sensitivity, different rates are selected; details of the specific parameters used are described in Section 4.2. The throughputs of these two different polymers are tested at various temperature profiles, ranging from 180 °C to 280 °C. The initial (P_i_) and the after-stabilisation (P_∆_) die-head pressures achieved under these different processing parameters are recorded. Pressure was deemed to be stable once a constant value had been observed. The time deemed suitable for observed a stable pressure value was at least 15 min. Generally, the time at a given pressure and temperature was 30 min before the value was collected. At this point, the pressure value was recorded and is presented in this study.

### 3.2. Extrusion Throughput Efficiency

To thoroughly evaluate the influence of distinct spinneret designs on polymer extrusion efficacy, a series of throughput efficiency tests are conducted using PP at 220 °C with a polymer output of 20 mL/min. To track material flow, 2 gr of blue dye (medical blue m/b in polyolefin-based (46004 16-E, Ampacet Corp., Tarrytown, NY, USA)) is introduced into the hopper.

Two key parameters were monitored throughout this testing process: residence and purge times. Residence time is the interval between the addition of the dye into the feed system and its initial visual detection in the extrudate, thereby indicating the duration that the polymer spends within the extrusion system. The purge time is measured as the point at which the dye is no longer noticeable in the extrudate, serving as an indicator of the system’s efficiency in material changeover and the presence of potential ‘dead spots’ (i.e., areas within the extruder or die where the polymer can stagnate). It is noted that the dye–tracer method employed in this study provides an estimate of the mean residence time and the overall residence-time distribution, rather than the minimum residence time of the polymer within the extrusion system. Consequently, conclusions regarding the presence of ‘dead spots’ are not drawn from the initial appearance of the dye, but from the purge time and persistence of the tracer, which reflect long-tail residence behaviour associated with stagnant or low-velocity regions.

## 4. Results and Discussion

### 4.1. Computational and Parametric Analysis

The simulation results highlight significant discrepancies among different spinneret designs. The kinked entry design (D1) shows a significant reduction in pressure drop, while the inverted dome design (D5) minimises dead zones, leading to more uniform flow. Shear rate data ranges from 500 to 700 s^−1^, depending on the design, with maximum velocities observed near the exit regions of the spinneret. Exit pressure and velocity varies across designs, emphasising the importance of each design’s geometry on extrusion performance. Rounded edges (D10) show a reduction in dead zones, while the reduced volume filter (D3) and smaller dome sizes (D8) lead to a more uniform velocity distribution. Detailed comparisons reveal that designs like D15, with a low kinked entry, achieve higher pressure stability at various flow rates.

A comparison between the maximum pressure, shear rate, and velocity achieved for the different configurations is given in Table 2. The maximum shear rate is given globally and relative to the midplane cross-section, as shown in Figure 1b. From the table, the original configuration (D0) has the highest speed. Having a cooler spinneret (D13) has a negligible effect on velocity and shear rate compared to the original design, while it increases pressure. The double layer (D4) exhibits a significant drop in pressure and speed due to the increase in spinneret holes, while the shear rate remains constant.

**Figure 4:**
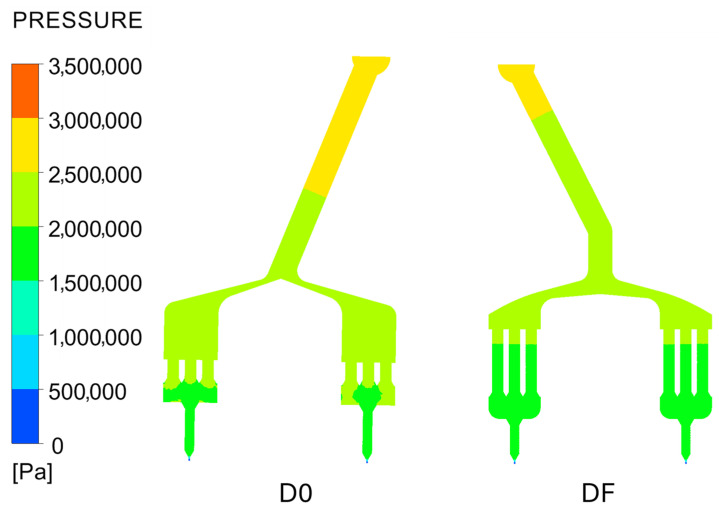
Comparison of pressure distributions between the original design (D0) and the final spin pack design (DF).

The comparison of various spinneret designs demonstrates that each geometry has a distinct influence on flow uniformity and extrusion quality. From this analysis, a ”final” design (DF) was developed, combining features from D3 (reduced volume filter), D8 (smaller dome size), D10 (rounded edges), and D15 (kinked entry). This new design is compared against the baseline design (D0). In Figure 4, the pressure profiles of DF and D0 are compared, showing a significant reduction in pressure for DF.

The combined effect of these improvements is a notable drop in die-head pressure for DF compared to D0 (experimentally observed to be ~5–20% lower). This aligns with general findings in polymer extrusion design: optimizing flow paths and removing sharp obstructions can improve throughput and reduce required pressure [22]. For instance, Liang et al. reported that a smoother die entry angle yields a lower pressure drop, highlighting how geometric optimization cuts flow resistance [23] Similarly, Fraunhofer researchers demonstrated that redesigning spin pack cavities to eliminate dead zones produces more uniform wall shear stress and pressure distribution, thereby enhancing flow while only moderately affecting pressure requirements [24]. In DF, because the main pressure drops occur at the fine capillaries and filter (which are now optimized), the two large cavities (filter chamber and distribution dome) contribute only minimal pressure loss [16]. The pressure profile measurements (Figure 4) indeed show that at every point in the spin pack, DF’s pressure is lower than D0’s—a direct consequence of reduced flow resistance in the new design.

**Figure 5:**
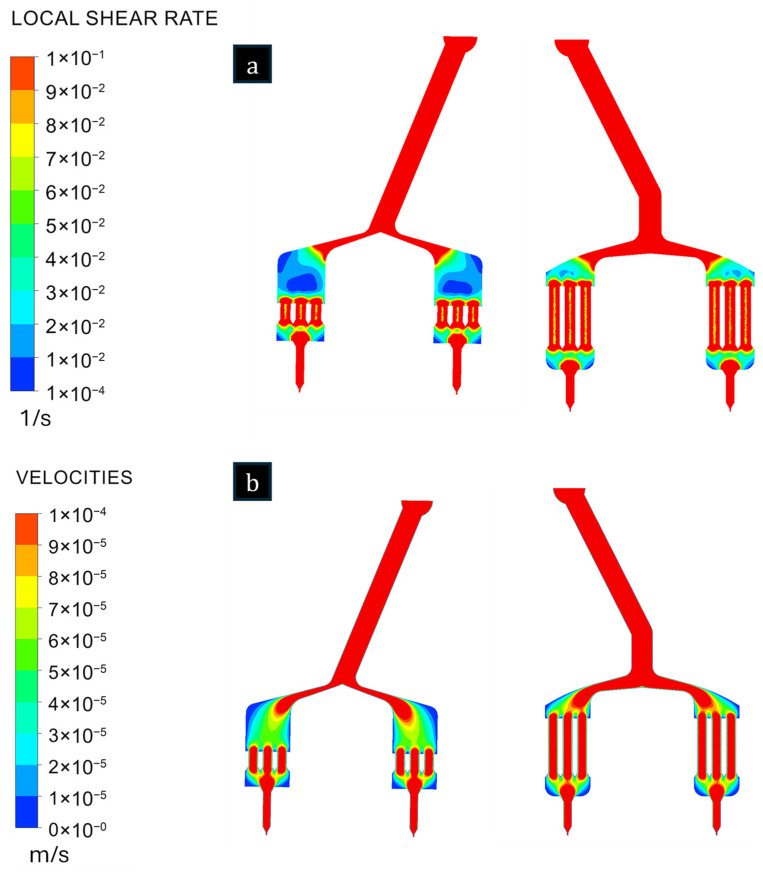
Comparison of shear rate (**a**) and velocity distributions (**b**) between the original design (D0) and the final design (DF) in the cross-sectional plane.

In Figure 5, the shear rate and velocity profiles are also compared, with DF exhibiting a more uniform velocity distribution and fewer regions of low velocity. As shown in Table 3, the maximum values for shear rate, pressure, and velocity are compared between the two designs. The results indicate that DF has a reduction in maximum pressure and velocity, accompanied by a slight increase in maximum shear rate, compared to D0.

The significant reduction in average residence time and the elimination of long-tail residence-time outliers in our DF spin pack are consistent with the findings of Leithäuser et al., who showed that removing dead zones through optimization of the spin-pack cavity geometry results in low and uniform residence-time profiles, which are critical for preventing material degradation [16].

### 4.2. Experimental Validation of Novel Spinneret Design

**Figure 6:**
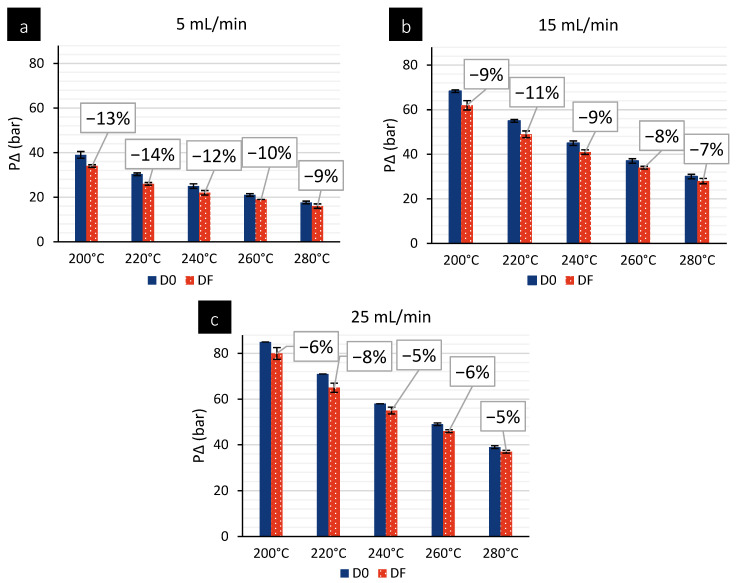
Graphical comparison of die-head pressures recorded at various temperature profiles for (**a**) 5 mL/min, (**b**) 15 mL/min, and (**c**) 25 mL/min metering pump-flow speeds using PP.

To validate the final spin pack design proposed by our numerical campaign, the DF design is manufactured. The DF spin pack design consists of a spinneret with 24 holes (0.4 mm Ø × 1.0 mm length). A comparable 24-hole spin pack is used as a benchmark for this study. Firstly, the two different spin pack designs are tested using PP at temperatures ranging from 180 °C to 280 °C, with polymer flows of 5, 15, and 25 mL/min. Figure 6 presents a graphical comparison of the different die-head pressures recorded during the different temperature profiles. Data represent the mean of three experimental iterations, presented along with their corresponding standard deviations. At 180 °C, the extruder encounters difficulties feeding the polymer, possibly due to the low temperature, which results in some of the polymer not melting adequately. This leads to irregular flow behaviour, and compromises material homogeneity at the die. Consequently, data obtained under these conditions are deemed non-representative and are excluded from the final analysis. Results reveal that the DF spin pack exhibits die-head pressures approximately 5% to 20% lower than those of its counterpart, with this disparity becoming more pronounced at temperatures ranging from 180 °C to 240 °C. These lower die-head pressures can be correlated with reduced polymer flow resistance at the die head, highlighting the effectiveness of the novel spin pack design in improving polymer throughput compared to its counterpart under comparable processing conditions. The combined effect of these improvements is a notable drop in die-head pressure for DF compared to D0 (experimentally observed to be ~5–20% lower). This aligns with general findings in polymer extrusion design: optimizing flow paths and removing sharp obstructions can improve throughput and reduce required pressure [22].

**Figure 7:**
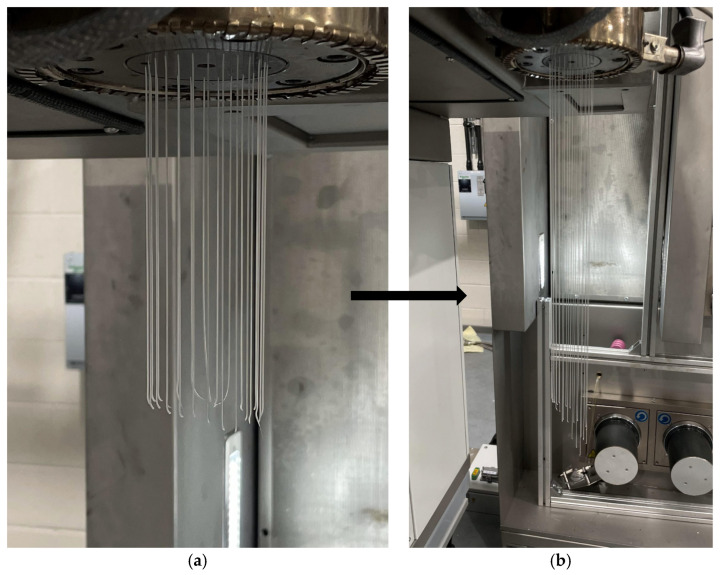
Comparisons of the extrudate observed during the testing of the D0 spin pack using TPU at 200 °C. (**a**) D0 spin pack initial extrudate at 200 °C; (**b**) extrudate thereafter.

**Figure 8:**
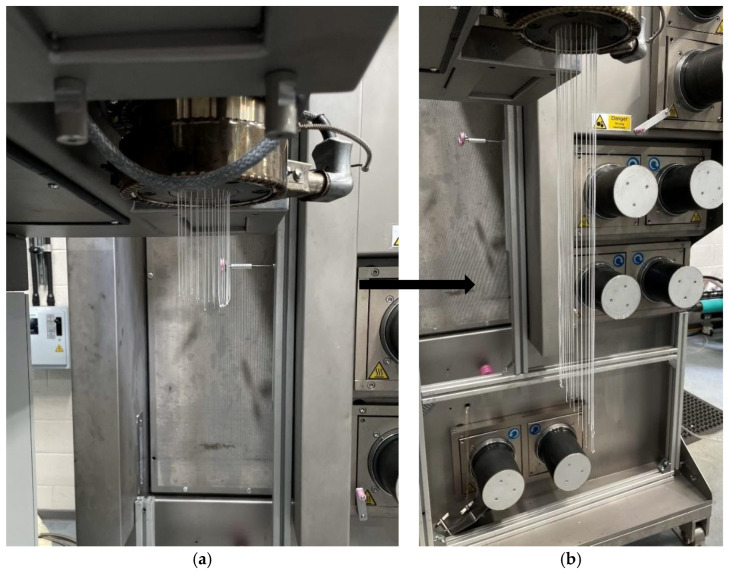
Comparisons of the extrudate observed during testing of DF spin pack extruding TPU at 200 °C. (**a**) DF spin pack initial extrudate at 200 °C; (**b**) extrudate thereafter.

Similarly, spin packs are tested using TPU at 190 °C, 195 °C, and 200 °C, with polymer throughput rates of 5, 8, and 15 mL/min. Different temperature profiles and polymer throughputs are considered, taking into account the thermal sensitivity of TPU. It is hypothesised that using a more thermally sensitive and less viscous polymer (MFI 17) would reveal more evident differences in polymer flow behaviour within the die. Similar to previous trends, the DF spin pack exhibits lower die-head pressures compared to its counterpart. Using both spin packs, it is observed that the extrudate initially appeared even as it exited the spinneret face; however, after a few seconds, it began to tilt forward, as shown in Figure 7 and Figure 8. This behaviour is likely to occur due to two possible reasons. First, there are likely localised cold spots in the heated collar around the spin pack. Uneven temperatures can increase viscosity non-uniformly, causing the melt to flow unevenly [25,26]. Secondly, with shear-thinning polymers such as PP or TPU, wall slip can occur at the die walls, resulting in asymmetric flow or an angular exit from the spinneret [27]. In future work, further CFD studies could be conducted to minimise this uneven polymer flow. By varying further parameters (thermal uniformity, polymer velocities and spinneret hole size) this extrudate tilting could be minimised further, ensuring even flow throughout the whole spin pack.

### 4.3. Extrusion Throughput Efficiency Tests

**Figure 9:**
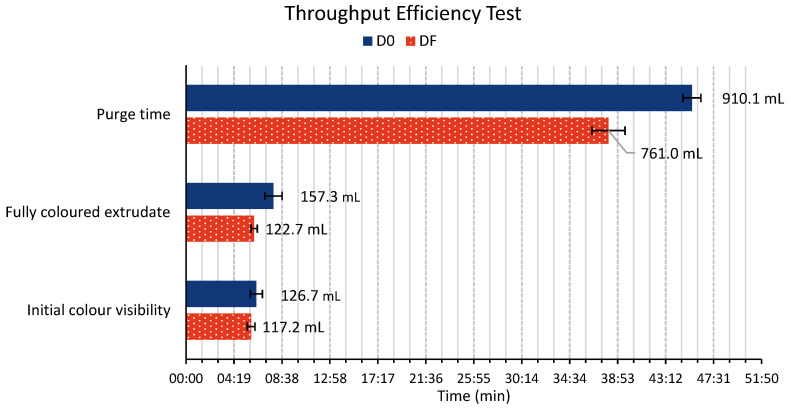
Throughput efficiency test comparing the D0 and DF spin packs. Initial colour visibility, full-colour extrudate, and purge time are reported both as processed polymer volume (mL) and corresponding time (min).

**Figure 10:**
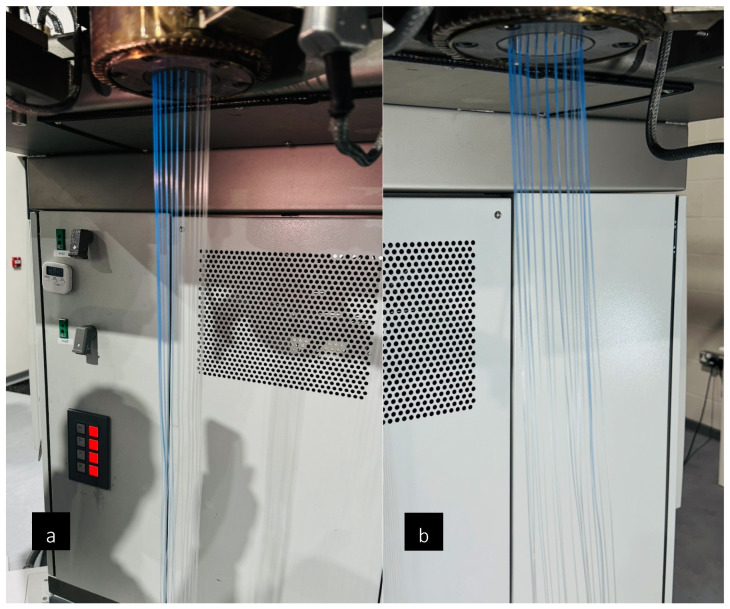
Flow-uniformity differences revealed by dye-tracer visualization during efficiency throughput tests of (**a**) the D0 spin pack and (**b**) the DF spin pack.

The extrusion throughput efficiency test results are illustrated in Figure 9. Two main parameters are evaluated: the residence time and the purge time. Notably, the DF spin pack design demonstrates shorter residence and purge times compared to its counterpart. Overall, the DF design proves to be 16% more efficient than the D0 design. The reduced purge time is particularly significant, and suggests that the DF spin pack’s design effectively minimises potential ‘dead spots’ where the polymer could become trapped. Furthermore, this dye-tracer method exposed uneven melt flow when using the D0 spin pack compared to the DF spin pack, as shown in Figure 10, revealing stagnant or low-velocity regions. These results highlight its superior design in facilitating a more efficient and complete material flow through the extrusion system. The significantly shorter purge time of the DF spin pack indicates minimization of dead zones. Dead spots in polymer melt equipment are known to trap degraded material, prolong purging, and compromise product quality. Streamlined geometries that eliminate sharp corners and stagnation pockets reduce residence time, improve purging efficiency, and enhance polymer flow-through [16,24].

## 5. Conclusions

In this study, a novel spin pack design aimed at improving polymer extrusion performance is introduced. A comprehensive CFD parametric investigation is carried out to optimise the geometry of the baseline spinneret (D0) and to develop an enhanced configuration capable of promoting smoother and more uniform polymer flow. The resulting design (DF) demonstrates a substantial reduction in dead zones within the die, a more uniform velocity distribution, and a marked decrease in regions of elevated shear when compared with the standard configuration.

Experimental validation further confirms that the improved spin pack requires lower extrusion pressures across a range of polymers and processing conditions. It also significantly mitigates polymer stagnation, as shown by shorter residence times and faster purging cycles, both indicators of more uniform flow and greater overall process efficiency. These combined findings highlight the potential of the redesigned spin pack to enhance extrusion performance by enabling systematic geometry optimization that directly links flow uniformity and residence-time reduction to measurable processing benefits.

Ongoing and future work will incorporate more advanced numerical modelling to explore in greater depth the mechanisms responsible for melt non-uniformity at the spinneret exit. Moreover, we will explore the adoption of the lattice Boltzmann method [28] as a complementary simulation framework, exploiting its intrinsic locality and scalability to enable high-fidelity, geometry-resolved analysis of polymer melt flow and residence-time distributions in advanced spinneret designs.

## Figures and Tables

**Table 1:** Rheological parameters for the polypropylene (PP) melt taken from Ansys Polyflow 2024 R2.

Parameter	Symbol	Value	Unit
Zero-shear viscosity	η_0_	1.522 × 10^4^	Pa×s
Infinite-shear viscosity	η_∞_	0	Pa×s
Relaxation time	*λ*	0.1309	s
Power-law index	*n*	0.1269	–
Yasuda exponent	*a*	0.3669	–

**Table 2:** Comparison of pressure (bar), shear rate (s^−1^), and velocity (m/s) for D0–D15 designs.

Name	Description	Pressure (bar)	Shear Rate (s^−1^)	Velocity (m/s)
D0	Original	33.9	564.1–471.3	0.051
D1	Kinked entry high	47.3	599.3–529.5	0.042
D2	Bi-Co Version of deep entry	25.5	592.8–511.9	0.042
D3	Reduced Volume Filter	29.3	600.7–510.3	0.042
D4	Double layer (48 holes) Spinneret	22.2	534.8–516.2	0.020
D5	Inverted Dome	26.0	583.8–490.6	0.042
D6	No Dome	25.6	596.5–500.8	0.044
D7	Offset entry	33.1	664.7–509.4	0.045
D8	Smaller dome size	27.3	590.3–508.3	0.044
D9	Pyramid shape	26.5	585.7–505.3	0.044
D10	Rounded edges	26.8	586.3–510.7	0.044
D11	Wider cone	26.4	610.1–541.2	0.042
D12	Lipped cone	27.5	593.0–500.0	0.044
D13	Cooler spinneret	37.4	568.9–473.4	0.050
D14	Doughnut cone	27.5	591.5–512.8	0.044
D15	Kinked entry low	33.9	590.7–518.9	0.044

**Table 3:** Comparison of pressure, share rate, and velocity for D0 and DF spinneret designs.

Name	Pressure (bar)	Shear Rate (s^−1^)	Velocity (m/s)
D0	30.2	569.93	0.051
DF	26.9 (−11%)	680.96 (+19%)	0.044 (−14%)

## Data Availability

The original contributions presented in this study are included in the article. Further inquiries can be directed to the corresponding authors.
